# The Screening Research of Anti-Inflammatory Bioactive Markers from Different Flowering Phases of *Flos Lonicerae Japonicae*

**DOI:** 10.1371/journal.pone.0096214

**Published:** 2014-05-08

**Authors:** Min Jiang, Yan-qi Han, Meng-ge Zhou, Hong-zhi Zhao, Xue Xiao, Yuan-yuan Hou, Jie Gao, Gang Bai, Guo-an Luo

**Affiliations:** 1 State Key Laboratory of Medicinal Chemical Biology and College of Pharmacy, Nankai University, Tianjin, People’s Republic of China; 2 Tianjin Key Laboratory of Molecular Drug Research, Nankai University, Tianjin, People’s Republic of China; 3 Department of Chemistry, Tsinghua University, Beijing, People’s Republic of China; University of North Dakota, United States of America

## Abstract

*Flos Lonicerae Japonicae* (FLJ) is an important cash crop in eastern Asia, and it is an anti-inflammatory Traditional Chinese Medicine. There are large variations in the quality of the marketed FLJ products. To find marker ingredients useful for quality control, a tandem technology integrating ultra-performance liquid chromatography/quadrupole time-of-flight mass spectrometry (UPLC-Q/TOF), principal component analysis (PCA), heat map analysis and hierarchical cluster analysis coupled with a NF-κB luciferase reporter gene assay were used to identify the different ingredients from the green bud, white bud, flowering stage and leaf stages, as well as to screen the anti-inflammatory activity of FLJ compositions. As flowering progressed, the anti-inflammatory effects of FLJ gradually decreased; however, chlorogenic acid, swertiamarin and sweroside should be used to evaluate the quality of FLJ products.

## Introduction

Flos Lonicerae Japonicae (FLJ) refers to the flower buds [1] of *Lonicera japonica Thunb*, which is also known as honeysuckle. FLJ is native to eastern Asia [2], including China, Japan, and Korea. FLJ is used as a traditional Chinese medicine; the first medicinal use of FLJ was recorded in the Compendium of Materia Medica (A.D. 1596) and is officially listed in the Chinese Pharmacopoeia (2010 version). Currently, FLJ has found wide use in herbal drugs, health products and food. In 2010, FLJ had an 800,000-ton domestic demand in China, and the economic benefits of this demand exceeded 1 billion United States dollars [3]. Generally, the flowering phase of FLJ has been divided into the three stages: the green bud stage (Bud size 1.0–3.5 cm, green), the white bud stage (Bud size 3.0–4.5 cm, white) and the flowering stage (Blooms). All of these stages of FLJ are commercially available. Currently, identifying the appropriate stages for harvesting FLJ remains controversial. In the Chinese Pharmacopoeia (2010 version), only two components (chlorogenic acid and cynaroside) were used to evaluate the quality of FLJ products. However, FLJ contains a complex mixture of numerous chemical compounds; therefore, measuring two particular constituents remains insufficient when evaluating the quality of FLJ products. Due to these shortcomings, the quality of FLJ on the market remains variable. Validating the picking period and the quality control standards are the current critical issues.

According to the Chinese Pharmacopoeia (2010 version), FLJ is used to treat furuncle carbuncle, pharyngitis, dysentery, erysipelas, colds, and other afflictions [4]. All of these diseases are related to inflammation. Inflammation is an important physiological response associated with numerous human diseases that can occur in any part of the body [5], [6]. FLJ has antifebrile, antimicrobial, and excellent anti-inflammatory effects [7]–[11]; its anti-inflammatory mechanism has been reported [7], [12]. FLJ exhibits a suppressive effect on the NO production during an *in vivo* air pouch model [10] and confirmed *in vivo* anti-inflammatory activity. Ryu and co-workers [11] have produced a highly purified FLJ extract; intravenously injecting this extract into mice produces anti-inflammatory and analgesic effects by inhibiting cylcooxygenase-2 induction, inducible nitric oxide synthase, and 5-lipoxygenase activities.

There are few reported comprehensive surveys of the anti-inflammatory compounds in FLJ. Therefore, revealing the pharmacodynamic materials in FLJ is urgent. The traditional method for screening bioactive components requires extracting and isolating purified compounds and using pharmacological models to detect, isolate, and structurally elucidate the biologically active constituents. This method consumes a great deal of time and material, making it inefficient for directly screening bioactive compounds. Therefore, a rapid and effective anti-inflammatory activity screening assay for FLJ is necessary [13].

In this study, a tandem method integrating UPLC-Q/TOF-MS, PCA, heat map analysis, and hierarchical cluster analysis with a NF-κB luciferase reporter gene assay was used to identify the different components from the green bud, white bud, flowering stage and leaf stages, as well as to screen the anti-inflammatory activity of the compounds from FLJ.

## Materials and Methods

### 1. Ethics Statement

This study was approved by the Institutional Review Board at the Nankai University. This field study did not involve any endangered or protected species, and all the samples of FLJ we purchased were common species in China. And no specific permission was required.

### 2. Reagents and Chemicals

HPLC grade acetonitrile was purchased from Fisher Scientific (Pittsburgh, PA, USA). HPLC grade water was prepared from distilled water using a Milli-Q system (Millipore Laboratory, Bedford, MA, USA). Swertiamarin, sweroside and chlorogenic acid were purchased from Yifang S&T (Tianjin, China). Tumor necrosis factor-α (TNF-α) was obtained from PeproTech (Rocky Hill, NJ). All reagents for cell culture were purchased from GibcoBRL Life Technologies (Rockville, MD, USA). The Lipofectamine 2000 transfection reagent was obtained from Invitrogen (Carlsbad, CA, USA). Any additional reagents used in this study were analytical grade.

### 3. Herbal Materials

Thirty-two samples from FLJ, including the green bud (GB), white bud (WB), flower (FL) and leaf of FLJ (LE) (samples No. 1–8 corresponded to GB, samples No. 9–16 corresponded to WB, samples No. 17–24 corresponded to FL and samples No. 25–32 corresponded to LE), were purchased from Anguo in the Hebei province of China and identified by Professor Tiejun Zhang from the Tianjin Institute of Pharmaceutical Research. The chlorogenic acid and cynaroside contents were determined using the method described in the Chinese Pharmacopoeia (2010 version). All samples were powdered and passed through a 100-mesh sieve before extraction.

### 4. Cell Culture

Human embryonic kidney 293 (HEK 293) cells and human bronchial epithelial (BEAS-2B) cells obtained from the American Type Culture Collection (Rockville, MD) were cultured in DMEM and DMEM/F12 (Hyclone, USA), respectively, containing 10% fetal bovine serum (FBS) (Hyclone, USA), 100 U/mL penicillin and 0.1 mg/mL streptomycin at 37°C under 5% CO_2_ in an incubator.

### 5. Sample Preparation for Analysis

The dried, ground materials (1 g) were extracted with 10 mL of water using an Ultrasonic cleaner (40 kHz, 500 W, Ningbo, China) for 60 min at room temperature. The extracts were centrifuged at 3500 rpm for 10 min, and the supernatants were sequentially extracted with petroleum ether, dichloromethane, ethyl acetate and aqua-saturated n-butanol. Each fraction was evaporated under vacuum, yielding petroleum ether (PEE), dichloromethane (DME), ethyl acetate (EAE), n-butanol (BUE), and aqueous (AQE) extracts. The extracts were dissolved in 0.1% DMSO DMEM medium and used for the NF-κB luciferase activity studies. The residues were dissolved in water and filtered through a 0.22-µm membrane filter before UPLC-MS analysis.

### 6. Luciferase Reporter Assay System for NF-κB Inhibition

Before the experiments, the cells were treated with fresh serum-free medium for 24 h. Afterward, the HEK 293 cells were co-transfected with the NF-κB luciferase reporter plasmid pGL4.32 at 100 ng/well and the Renilla luciferase reporter vector plasmid pRL-TK at 9.6 ng/well for 24 h. Lipofectamine 2000 was used during the transfection according to the manufacturer’s instructions. After transfection, the cells were pre- incubated with the drugs for 1 h before being stimulated with 5 ng/ml TNF-α for 6 h. Subsequently, the luciferase activity was assayed using a Luciferase Reporter Assay System (Promega, WI, USA) after the HEK 293 cells were washed and lysed according to the manufacturer’s instructions. The luminescence was assessed with a Modulus luminometer from Turner Biosystems (Turner Design, Sunnyvale, CA, USA), and the relative luciferase activity (firefly and Renilla luciferase) was used to normalize the differences in transfection efficiency.

### 7. Anti-inflammatory Activity Detection of various Flowering Phases and Leaf of FLJ

The green bud stage (GB) of FLJ was the most common material available. In this paper, the anti-inflammatory activity of various GB extractions was first measured using the NF-κB luciferase reporter gene assay. The most active extracts from the various flower and leaf phases of FLJ were used to detect the anti-inflammatory activity, and the different ingredients were analyzed using UPLC-Q/TOF MS coupled with PCA.

#### 7.1. UPLC-Q/TOF MS conditions

A Waters Acquity UPLC instrument system (Waters Co., USA) equipped with a photo diode array detector (DAD) was used for the FLJ analysis. The system was controlled by MassLynx 4.1 software (Waters Co., USA). The separations were performed on a Waters Acquity UPLC BEH C_18_ column (2.1 mm×100 mm, 1.7 µm) at 30°C. A gradient elution of acetonitrile (A) and 0.1% formic acid (B) was performed as follows: 0–5 min, isocratic of 2% A; 5–9 min, 2–8% A; 9–12 min, isocratic of 8% A; 12–15 min, 8–10% A; 15–18 min, 10–15%; 18–22 min, 15–20% A; 22–27 min, 20–100%. UPLC GB fractions were collected in a 96-deep-well plate every 0.5 min and evaporated to dryness in a vacuum drying oven. The residues were dissolved in 100 µL of cell culture medium for further bioactivity studies.

Mass spectrometry was performed with a Waters Q/TOF Premier Mass Spectrometer with an electrospray ionization system (Waters MS Technologies, Manchester, UK). The ESI-MS spectra were acquired in negative ionization mode. The conditions for ESI-MS analysis were as follows: the capillary voltage was set to 2.5 kV, the sample cone voltage was set to 30 V, the desolvation gas flow was set to 600 L/h at 300°C, the cone gas was set to 50 L/h, and the source temperature was 100°C. The Q/TOF Premier acquisition rate was 0.1 s, with a 0.02 s inter-scan delay. The MS spectra were acquired from 50 to 1500 Da. Leucine-enkephalinamide acetate (LEA) was used as the lock mass (*m*/*z* 555.2931 in ESI+ and 553.2775 in ESI-) at 200 ng/mL and 20 µL/min.

#### 7.2. PCA of the substances

PCA was used to differentiate the characteristic components of the different FLJ samples. UPLC-Q/TOF MS data were imported into Markerlynx, and the data from Markerlynx were directly imported into the Simca-P software (version 11.5, Demo, Umetrics, Umea, Sweden) to ensure a smooth formation process. PCA was used to distinguish the principal components (PCs). However, each substance was also identified in Markerlynx and analyzed according to the UV data, as well as the primary and secondary mass spectrometry data, relative to the literature values.

### 8. Analysis of Relative Content

The relative content of each component in each samples of FLJ was revealed by a heat map. The peak areas of the target compounds were exported from Markerlynx, followed by normalization. The samples were analyzed using the Cluster software (version 3.0, Cluster), and the results were visualized using Java Treeview (version 1.1.6 R2).

### 9. Hierarchical Cluster Analysis

The resemblance and variation of the compounds in FLJ at different stages were evaluated by a hierarchical clustering analysis (HCA) performed based on the characteristics of the peak areas of the NF-κB inhibitor and marker profiles (total of 10 compounds). The peak areas of samples 1–32 formed a 32×10 matrix. The distances between the 32 samples were calculated using SPSS software (version 18.0, SPSS).

### 10. Determination of the Swertiamarin, Sweroside and Chlorogenic Acid Contents in FLJ

The determination of the swertiamarin, sweroside and chlorogenic acid contents was performed according to the UPLC conditions described above. The ultraviolet detector was set at 327 nm for chlorogenic acid and at 236 nm for swertiamarin and sweroside, respectively.

### 11. Confirmation of the Bioactivity of the NF-κB Inhibitors on BEAS-2B

The confirmation method for BEAS-2A was the same as that for the HEK 293 cells. Swertiamarin, sweroside and chlorogenic acid were added to the cells at different concentrations (100, 10, 1 µmol/L). The NF-κB inhibition of the three compounds was evaluated with a Luciferase Reporter Assay System, and the release of IL-6 and IL-8 into the BEAS-2B cell supernatants after stimulation by drugs or TNF-α was measured using an ELISA kit (Pierce/Endogen) according to the manufacturer’s instructions.

### 12. Statistical Analysis

The results are expressed as the standard error of the mean (SEM). The statistical analysis of the data over multiple comparisons was performed using a one-way ANOVA followed by the Bonferroni post-hoc test. For single comparisons, the significant differences between the means were determined using Student’s *t*-test. The statistical significance was set at *P<*0.05.

## Results

### 1. The NF-κB Inhibitory Effects of FLJ Extracts

**Figure 1:**
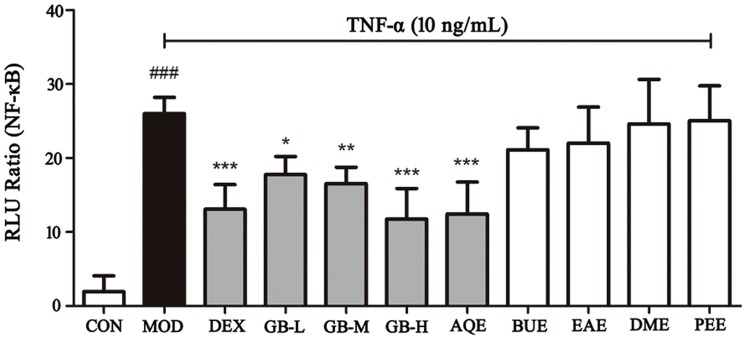
Effects of the extracts from FLJ on NF-κB levels. The values are presented as the means ± SEM; n = 6 for each group. One (*), two (**) or three (***) indicate *p*<0.05, *p*<0.01 or *p*<0.001 *vs* the TNF-α treated group, respectively; ### indicates *p*<0.001 *vs* the control group.

The effects of different extracts of the FLJ GB stage and its supernatant on NF-κB were evaluated at the cellular level using the luciferase reporter assay system. As shown in Fig. 1, the positive dexamethasone (Dex) (10^−5^ mol/L), low GB dose (GB-L, 0.1 mg/mL), middle GB dose (GB-M, 0.3 mg/mL), high GB dose (GB-H, 1.0 mg/mL) and AQE of FLJ (0.3 mg/mL) groups significantly inhibited the TNF-α induced NF-κB production (*P<*0.01). Consequently, the AQE extraction contained potential NF-κB inhibitors. Therefore, the AQE extractions of the four FLJ groups were used during further experiments.

### 2. Evaluation of the Bioactivity of the NF-κB Inhibitors

**Figure 2:**
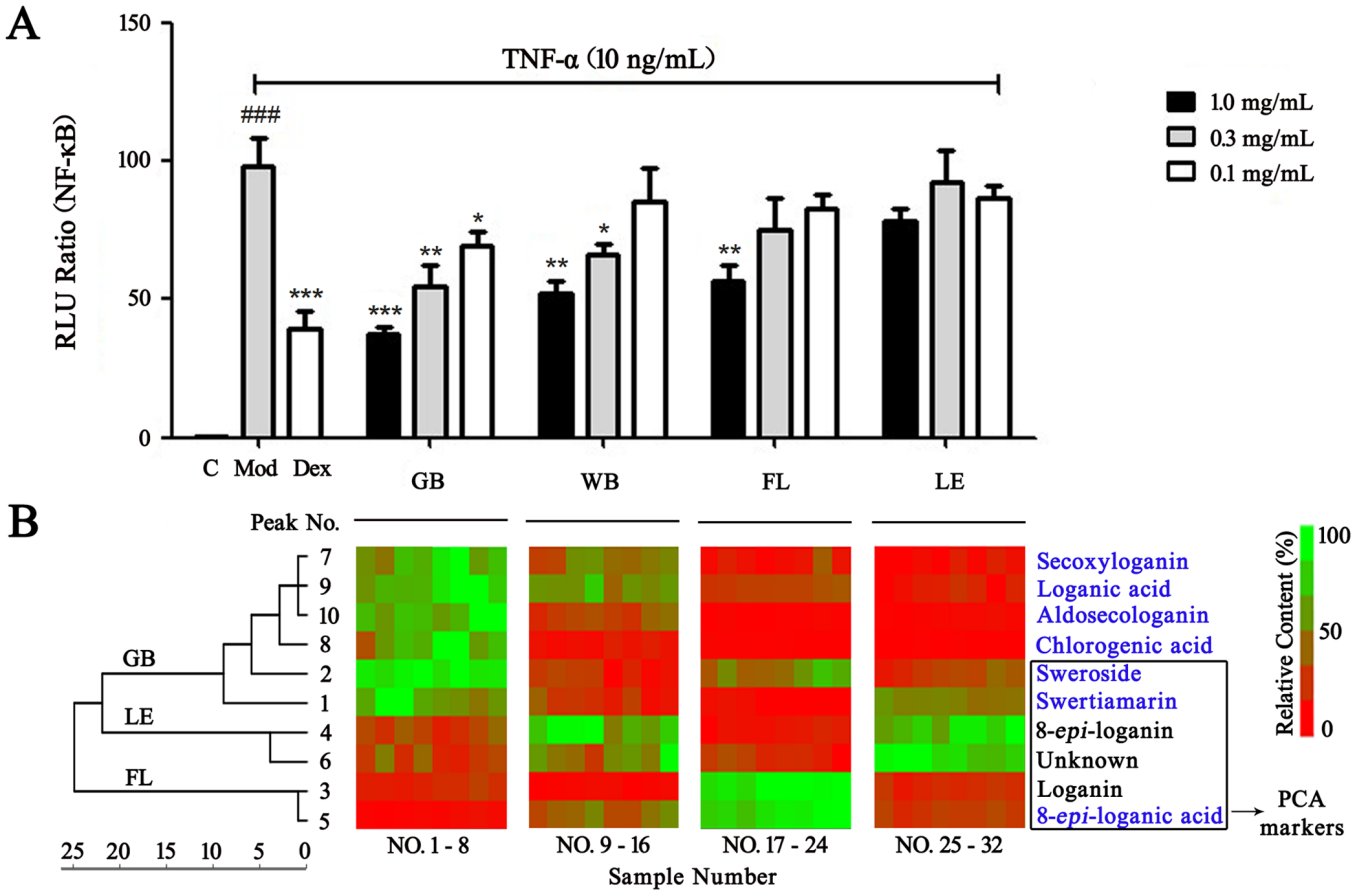
Effects of the different FLJ flower bud stages on NF-κB inhibition (A), the heat map and hierarchical cluster analysis of FLJ. A: The expression of NF-κB in TNF-α stimulated HEK 293 cells. The values are presented as the means ± SEM; n = 6 for each group. One (*), two (**) or three (***) indicate *p*<0.05, *p*<0.01 or *p*<0.001 *vs* the TNF-α treated group, respectively; ### indicates *p*<0.001 *vs* the control group. B: The relative contents of the marker ingredients and NF-κB inhibitors in different FLJ flower bud stages. Zero content was expressed as red and the largest content (100%) as green.

To evaluate the effects of the four FLJ groups on NF-κB inhibition, the AQE extracts for each stage were added to the TNF-α stimulated HEK 293 cells for the bioactivity tests using a NF-κB activity luciferase reporter assay system. As shown in Fig. 2A, for the low (0.1 mg/mL), middle (0.3 mg/mL) or high dose (1.0 mg/mL) groups, the FLJ GB demonstrated the highest inhibition of NF-κB expression. The FLJ WB exhibited decreasing anti-inflammatory activity, but the FLJ LE did not inhibit NF-κB. Therefore, the FLJ GB stage contained an anti-inflammatory agent operating at the cellular level; this result was consistent with its use in TCM. Therefore, the FLJ GB stage was used to separate and screen the NF-κB inhibitors via UPLC-Q/TOF MS analysis.

### 3. PCA Results and Identification of Marker Ingredients

**Figure 3:**
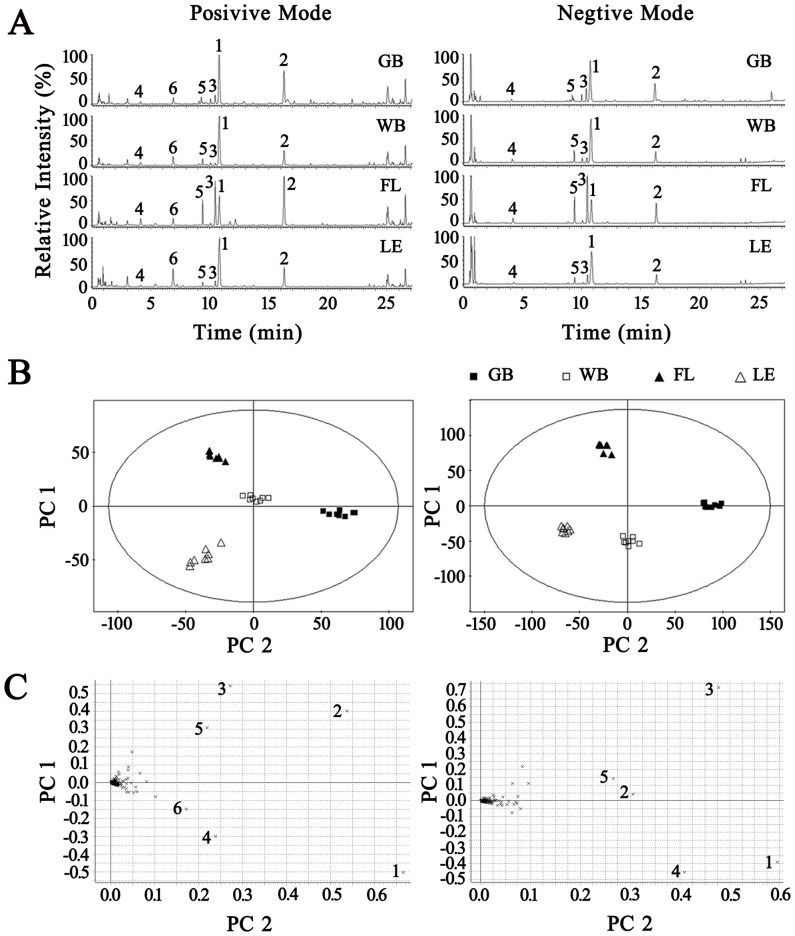
PCA and the BPI chromatograms at different FLJ flower bud stages. BPI chromatograms (A), score plots (B) and loading plots (C) in positive and negative ESI modes are shown. The peak numbers, significance values and relative contents (MAX.) are consistent with those in Table 1.

**Figure 4:**
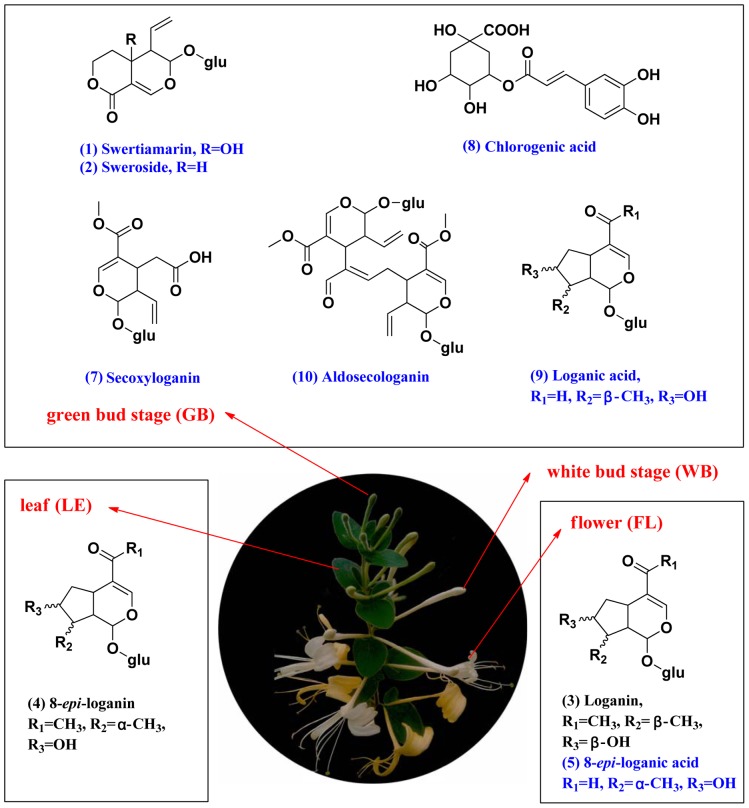
A picture of different flower bud stages of FLJ and the chemical structures of the marker ingredients and bioactive compounds in FLJ.

To evaluate the influence of sample preparation on the data, six parallel samples were prepared using the same preparation protocol; the retention times of the peaks remained almost unchanged, and the relative standard deviations (RSD) of the peak intensities of the major signals were below 3.9%. The precision of injection was studied by continuously analyzing six injections from the same samples. The RSDs of the peak areas were between 2.1 and 3.6%. The basic peak ion (BPI) current chromatograms from the different FLJ stages in both negative and positive ESI modes are presented in Fig. 3A. Obvious discrimination between the different FLJ stages was observed in the PCA score plots (shown in Fig. 3B) that were used to distinguish the components at the different stages based on their chemical fingerprints. All of the samples were classified into four groups, and none of them were misclassified, demonstrating that the constituents of the samples from different stages were significantly different. The loading plots were utilized to reveal the contribution of each principal component during our experiment. The distance from the origin of the loading plots to the marker demonstrated the significance of the marker, and a higher value indicates a more significant marker. From Fig. 3C, six compounds (swertiamarin, sweroside, loganin, 8-*epi*-loganin, 8-*epi*-loganic acid and an unknown component) contributed strongly to the clusters (significance value >0.1) in both positive and negative mode; the six components were therefore marker ingredients. The detailed results and structures of the marker ingredients are presented in Table 1 and Fig. 4, respectively.

**Table 1:** MS/MS data from (+/−) ESI-MS and identification of the PCA results and the bioactive compounds of FLJ.

PeakNo.	t_R_ (min)	Mode	MS[M-H]^−/^[M+H]^+^	MS/MS (m/z)	Molecularweight (Da)	Molecularformula	Identification	Significance		Max
								Pos	Neg	
**1**	10.90	Pos	375.1171	397[M+Na]^+^, 213[M+H-Glc]^+^,195[M+H-Glc-H_2_O]^+^,177[M+H-Glc-H_2_O- H_2_O]^+^	374.1213	C_16_H_22_O_10_	swertiamarin	0.5884	0.5037	GB
**2**	16.37	Pos	359.1310	381[M+Na]^+^, 197[M+H-Glc]^+^,179[M+H-Glc-H_2_O]^+^,151 [M+H-Glc-H_2_O-CO]^+^	358.1264	C_16_H_22_O_9_	sweroside	0.4736	0.2185	GB
**3**	10.52	Pos	391.1138	413[M+Na]^+^, 373[M+H-H_2_O]^+^,229[M+H-Glc]^+^,211[M+H-Glc-H_2_O]^+^, 197[M+H-Glc-CH_3_OH]^+^	390.3874	C_17_H_26_O_10_	loganin	0.4292	0.6130	FL
**4**	4.15	Pos					8-*epi*-loganin	0.2712	0.4317	LE
**5**	9.44	Pos	377.1342	399[M+Na]^+^, 359[M+H-H_2_O]^+^,215[M+H-Glc]^+^,197[M+H-Glc-H_2_O]^+^, 179[M+H-Glc-H_2_O-H_2_O]^+^	376.1369	C_16_H_24_O_10_	8-*epi*-loganicacid	0.2682	0.2135	FL
**6**	6.75	Pos	282.1354	196, 178, 87	/	/	Unknown	0.1591	NS a (<0.1)	LE
**7**	9.07	Pos	405.1386	427[M+Na]^+^, 243[M+H-Glc]^+^,225[M+H-Glc-H_2_O]^+^,211[M+H-Glc-CH_3_OH]^+^	404.1319	C_17_H_24_O_11_	secoxyloganin	NS	NS	GB
**8**	9.27	Neg	353.0834	191[M-H-caffeoyl]^−^	354.0951	C_16_H_18_O_9_	chlorogenicacid	NS	NS	GB
**9**	10.05	Pos	377.1452	399[M+Na]^+^, 359[M+H-H_2_O]^+^,215[M+H-Glc]^+^,197[M+H-Glc-H_2_O]^+^,179[M+H-Glc-H_2_O-H_2_O]^+^	376.1369	C_16_H_24_O_10_	loganic acid	NS	NS	GB
**10**	22.04	Pos	759.2735	579[M+H-Glc-H_2_O]^+^,417[M+H-2Glc-H_2_O]^+^,	758.2633	C_34_H_46_O_19_	aldosecologanin	NS	NS	GB

a *NS:* no significance.

### 4. Identification of Hit Compounds for NF-κB Inhibitors in the GB of FLJ

**Figure 5:**
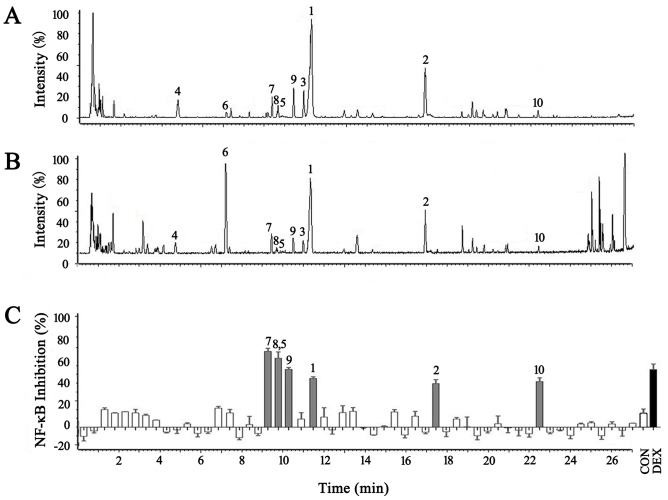
UPLC-Q/TOF coupled with a luciferase reporter assay to survey and identify NF-κB inhibitors in the GB of FLJ. A: BPI chromatogram in negative ESI mode. B: BPI chromatogram in positive ESI mode. C: The bioactivity chromatogram obtained via the luciferase reporter assay system for NF-κB inhibition. The peak numbers are consistent with those in Table 1.

To identify the effective compounds in FLJ, the AQE GB extract was separated using UPLC. The UPLC fractions were collected at 0.5 min intervals, and each fraction was tested for activity using the luciferase reporter assay system for NF-κB inhibitors. As shown in Fig. 5C, seven candidate compounds (peaks 1, 2, 5, 7, 8, 9 and 10) exhibited increased NF-κB inhibition twice that of the control. The potential NF-κB inhibitors could be classified into two types according to their chemical structures: chlorogenic acid and iridoid glycosides, including swertiamarin, sweroside, 8-*epi*-loganic acid, secoxyloganin, loganic acid, and aldosecologanin. Three NF-κB inhibitors from the GB belonged to the marker ingredients found in the different FLJ stages. The detailed results for the identified bioactive compounds and the MS and MS/MS information are listed in Table 1. The structures of the bioactive compounds in the AQE from the GB stage of FLJ are shown in Fig. 4.

### 5. Comprehensive Evaluation and Analysis

According to the above results, six marker ingredients were found in the different flower bud stages of FLJ, and seven bioactive components were identified in the GB stage of FLJ. These ingredients belonged to different chemical families and were common in the different stages of FLJ.

To reveal the relative content for each compound in each different stage, a heat map was established, as shown in Fig. 2B. The red color means “zero,” and green indicates “the largest” content of each ingredient based on the normalized data. The last six ingredients in the box were the marker ingredients identified by PCA analysis, and the components in blue were the NF-κB inhibitors revealed by the dual-luciferase reporter assay system. The six NF-κB inhibitors (swertiamarin, sweroside, secoxyloganin, chlorogenic acid, loganic acid, and aldosecologanin) were the most abundant in the FLJ GB stage, explaining why the GB stage exhibited the best anti-inflammatory activity. In addition, the contents of these six NF-κB inhibitors declined during the WB, FL and LE stages, which was consistent with their NF-κB inhibition abilities.

During the hierarchical clustering analysis using the Pearson correlation, the 10 key marker ingredients from the 32 batches of samples were divided into three main clusters (Fig. 2B): compounds 7, 9, 10, 8, 2 and 1 composed cluster one and were abundant in the GB stage; ingredients 4 and 6 were abundant in the LE stage and were grouped as cluster two; and ingredients 3 and 5 were abundant in the FL stage and were grouped as cluster three.

### 6. Determination of Swertiamarin, Sweroside and Chlorogenic Acid in FLJ

The swertiamarin, sweroside and chlorogenic acid contents were determined as representative bioactive compounds by UPLC. The linear ranges of swertiamarin, sweroside, and chlorogenic acid were 31.28 to 312.8 ng (Y = 56033X+289.41, r = 0.9997), 32.51 to 325.1 ng (Y = 30923.17X−6.69, r = 0.9997), and 33.2 to 397.44 ng (Y = 91679X+928.81, r = 0.9993), respectively. The average concentrations of swertiamarin, sweroside, and chlorogenic acid in the FLJ were approximately 12.30, 6.23 and 18.59 mg/g, respectively.

### 7. Confirmation of the Bioactivity of NF-κB Inhibitors from FLJ

**Figure 6:**
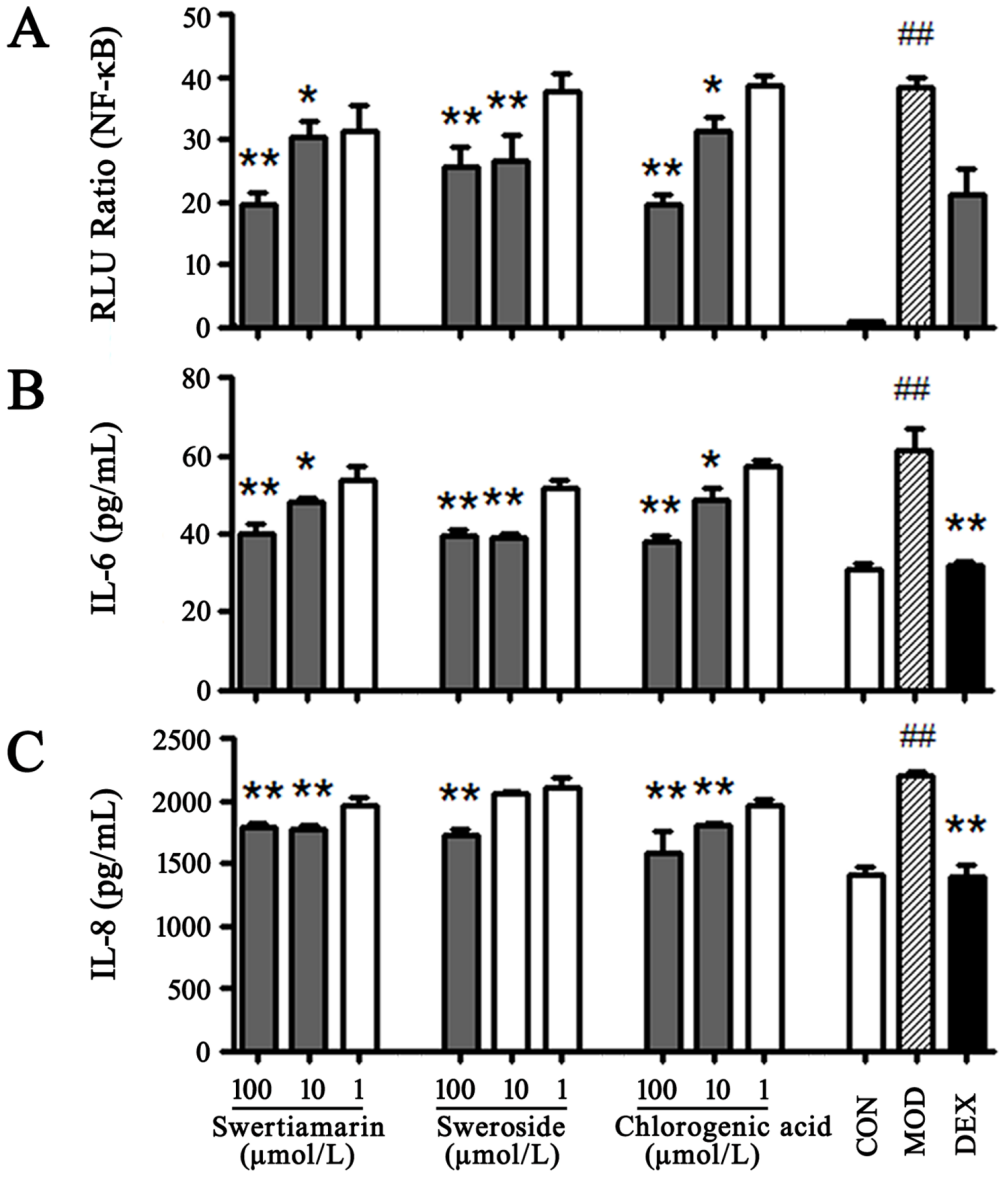
Confirmation of the effects by potential NF-κB inhibitors. A: Effects of the potential NF-κB inhibitors on the NF-κB expression. B and C: IL-6 and IL-8 expression in TNF-α induced BEAS-2B cells, respectively. Each bar represents the mean ± SEM; *n* = 6 for each group. One (*) or two (**) indicate *p*<0.05 or *p*<0.01 *vs* the TNF-α treated group, respectively; ## indicates *p<*0.01 *vs* the control group.

As shown in Fig. 6A, swertiamarin, sweroside and chlorogenic acid demonstrated significant NF-κB inhibition (*P<*0.05). The NF-κB transcription factors synergistically activate the transcription of the IL-6 and IL-8 inflammatory cytokines [14]. The above NF-κB inhibitors were further validated using an IL-6 and IL-8 release test. In this experiment, swertiamarin, sweroside and chlorogenic acid (1, 10, and 100 µmol/L) were added to TNF-α-stimulated BEAS-2B cells to determine whether these compounds efficiently inhibited the release of IL-6 and IL-8. As shown in Fig. 6 (B and C), the IL-6 level was decreased in the swertiamarin, sweroside, and chlorogenic acid-treated (10 and 100 µmol/L) groups (*P<*0.05). For IL-8, the swertiamarin, sweroside, and chlorogenic acid had significant suppressive effects (*P<*0.05). The positive control was Dex (10 µmol/L) and significantly inhibited both IL-6 and IL-8 release (*P<*0.01). These results confirmed the anti-inflammatory effects of different NF-κB inhibitors present in FLJ.

## Discussion

In the current study, an aqueous FLJ extract exhibited a similar anti-inflammatory efficacy to that of the full extract and was stronger than any other extract. This result is consistent with previous studies [9] and with FLJ clinical applications. Therefore, we carried out further experiments using aqueous extracts.

Currently, all three FLJ stages are commercially available. Our study revealed that these products were qualified according to the standards listed in the Chinese Pharmacopoeia (the content of Chlorogenic acid >1.5%, and cynaroside >0.05%). However, after studying the anti-inflammatory effect of the aqueous extract from the flowering and leaf stages of FLJ, the anti-inflammatory effect of the GB was proven to exceed the other stages. Therefore, detecting two components only, such as chlorogenic acid and cynaroside, cannot be used during a comprehensive assessment of the FLJ.

NF-κB is a transcription factor [15], [16] expressed in numerous cell types and is involved in the early immune and inflammatory responses. NF-κB also interacts with and regulates many molecules, including TNF-α, IL-1β, IL-2, IL-6, and IL-8. In our previous study, we developed a bioactivity-integrated UPLC-Q/TOF protocol for identifying NF-κB inhibitors [17], [18]. PCA is a multivariate statistical method used to distinguish chemical components using cluster analysis and a factorial k-means analysis of relatively large data sets acquired from HPLC-DAD, UPLC-MS and spectroscopic techniques [19], [20]. However, some components with a weak ion response, such as chlorogenic acid, cannot be identified as distinguishing ingredients. The heat map analysis and hierarchical cluster analysis can mitigate this deficiency. In this study, a tandem technology that integrates UPLC-Q/TOF-MS information with a NF-κB luciferase reporter gene assay was used to identify the distinguishing ingredients from four sample groups. Ten ingredients integrated from six PAC marker ingredients and seven bioactive components were found in the different flower bud stages of FLJ (Fig. 2). Cynaroside was not detected in the aqueous extracts due to its low content and poor water solubility. On the other hand, chlorogenic acid exhibited a stronger anti-inflammatory activity consistent with the previously reported data [21]–[23]. Although it was not identified as a distinguishing ingredient by PCA due to its weak ion response, the two other methods indicate that its content differs across the various stages. The different anti-inflammatory activity demonstrated by the various samples could also be explained by the different swertiamarin and sweroside contents. Previous studies reported that swertiamarin [24] possesses antiedematogenic effects compared to the three inflammatory agents used to induce paw edema. Swertiamarin also displays *in vitro* antioxidant activity against several free radicals. Sweroside [11] can mediate anti-inflammatory activities by suppressing inflammatory enzymes, such as COX-2, iNOS, and 5-lipoxygenanse (5-LOX). As effective ingredients, and excepting chlorogenic acid, swertiamarin and sweroside should be used to evaluate the quality of FLJ products. On the other hand, since FLJ had complex compound components, although some active ingredients showed NF-κB inhibitor activity had been found in this study, their synergistic mechanisms need an in-depth study.

## Conclusion

In conclusion, there are large variations in the quality of the FLJ products sold on the market. Our study revealed that the anti-inflammatory effect of FLJ gradually decreased as flowing progressed, the GB demonstrated the most bioreactivity, and six typical ingredients were found during the FLJ GB stage. Adding the analysis of the swertiamarin and sweroside contents during quality control could improve the regulation of FLJ products.
